# An Investigation into Major Sheep Diseases and Management Practices in North Shewa Zone, Oromia, Ethiopia

**DOI:** 10.1155/2022/4868391

**Published:** 2022-09-08

**Authors:** Tadesse Birhanu, Tesfaye Debelu, Said Muhammed, Fikiru Getachew

**Affiliations:** ^1^Salale University, College of Agriculture and Natural Resource, Department of Veterinary Science, P.O. Box: 245, Fiche, Ethiopia; ^2^Salale University, College of Agriculture and Natural Resource, Department of Animal Science, P.O. Box: 245, Fiche, Ethiopia

## Abstract

Sheep have many advantages over large ruminants for most smallholder farmers: lower feed costs, quicker turnover, easy management, and appropriate size at slaughter can be mentioned. They produce in a wide range of agroecologies, from arid lowlands to extremely cool highlands. However, their productivity is hindered by disease burden and poor management practices. In the study area, information on the disease of sheep and related management practices is lacking. Thus, the study aimed to determine the major sheep diseases and management practices in North Shewa Zone, Oromia, Ethiopia. A cross-sectional study design was used from October 2020 to July 2021 in the zone. A multistage sampling technique was used to select study districts and their respective kebeles, while the households were purposively selected. Questionnaire survey, in-depth interview, and physical clinical examination were conducted. A total of 400 households were involved in this study, a majority (32.8%) of whom were illiterates. Ovine pasteurellosis (55.8%) was the major bacterial disease in highlands, whereas sheep pox (54.5%) was the most challenging viral disease in the area. Mange mites (41.3%) were the major parasitic disease. The design of houses was medium (34.5%) which were bedded using sand floor (79.8%) and grass (5.75%), but the drainage system of the house was poorly designed (46.8%) in highlands. A majority of the owners (67.3%) used traditional medicines for the treatment of sheep disease. This study concluded that the burden of the diseases was higher and the management practices were poor in the area, deteriorating the economic benefit of farmers from sheep production. Thus, it urges for operating technical interventions.

## 1. Introduction

Ethiopia has a sheep population of 30.7 million which has an enormous contribution to cultural and social livelihoods and religious values for the large and diverse human population, is a means of investment, and earns foreign exchange to the nation [1]. Sheep have many advantages over large ruminants for most smallholder farmers such as lower feed costs, quicker turnover, easy management, and appropriate size at slaughter. Sheep can produce in wide range of agroecology from arid lowlands to extremely cool highlands. The animal plays a major economic role in the overall production system of large- and small-scale farmers, where most sheep production is for wool, leather, and meat production [2]. There is a huge and diverse sheep population which is a requisite to improve the livelihoods of the poor smallholder farmers. Sheep provide valuable contributions for smallholder producers via income generation, direct food sources, nonfood utilities, and various sociocultural privileges; especially during drought, they mitigate shortage of food [3].

The indigenous breeds of sheep account for about 99.6% of the total sheep populations which are reared under poor smallholder farmers and pastoralists under traditional and extensive production systems in Ethiopia. There is huge demand of live sheep and meat both in local and international markets [4]. At optimum off-take rates, Ethiopia can export 700,000 sheep annually and at the same time supply 1,078,000 sheep for the domestic market [5]. The skin and meat of Salale sheep have invaluable contribution to Ethiopia's national economy with high-quality international standards. There are smallholder sheep production systems where an extensive system is commonly practiced. Consequently, the productivity is hindered by different diseases, unimproved breed, and management practices [1].

A few studies that were conducted in different corners of the country indicated that sheep are mainly suffering from different infectious diseases such as sheep pox, ovine pasteurellosis, ovine brucellosis, heamonchosis, lung worm, anthrax, mange mites, tick infestation, and liver flukes which are the major health problems in the country. The studies were focused on the Abattoir survey which could not indicate the household level [6–10]. The great potential is also hindered by the traditional management system, inferior genetic makeup coupled with malnutrition, and absence of well-developed market infrastructure [11].

Although sheep provide valuable contributions for smallholder producers and national economy via income generation, direct food sources, nonfood utilities, and generating foreign currency from export of leather, live sheep, and meat; there was no well-documented information on sheep diseases and management practices in North Shewa Zone, Oromia, Ethiopia, which is crucial for the implementation of effective prevention and control strategies [12]. Therefore, this study was designed to elucidate fundamental information on the major sheep diseases and management practices in the zone.

## 2. Materials and Methods

### 2.1. Description of the Study Design and Areas

A cross-sectional study design was conducted from October 2020 to July 2021 in North Shewa Zone, Oromia Regional State, Central Ethiopia. The zone is located at 112 km north of Addis Ababa, the capital city of Ethiopia, on Addis Ababa to Gojam Main Road. It is located at latitude and longitude of 9°48′N 38°44′E and an elevation between 2,738 and 2,782 meters above sea level. The average minimum and maximum annual rainfall are 793 mm and 1443 mm, respectively, while the average minimum temperature is 10°C and maximum temperature is 32°C. It is bordered by Amhara National Regional State, West Shewa Zone, Finfinne surrounding special zone, and East Shewa Zone. The zone has 13 rural districts, one administrative town (Fiche), 18 towns, and 267 rural and 24 urban kebeles. Its altitude ranges from about less than 1000 m to over 3540 m. It has average annual rainfall ranging from 600–2000 mm. The average minimum temperature is 10°C and maximum temperature is 32°C [12].

The study was conducted in six selected districts (Kimbibit, Jidda, Girar Jarso, Degem, Kuyu, and Wuchale). Three potential sheep-producing kebeles were purposively selected from each district. Livestock production is the major production practices in the zone next to crop production which accounts for 1,676,748 cattle, 118,0430 sheep, 32,4274 goats, 106,472 horses, 8,035 mules, 270881 donkeys, 102,367 poultry, and 142,210 beehives (147,268 cultural, 9,101 intermediate, and 3,286 modern) in the zone. There are 240,000 sheep in Kimbibit; 146, 608 sheep in Jidda; 123,425 sheep in Wuchale; 80,715 sheep in Degem; 49,994 in Girar Jarso; and 49,269 sheep in Kuyu districts. Local sheep breed reared mostly under extensive and rarely semi-intensive production systems in the zone [12].

### 2.2. Sampling and Sample Size Determination

The sample size was calculated based on the formula of Bartlett et al. [13]. Lists of 15,140 smallholders' sheep producers were considered as the sampling frame (*N*).

(1) $$\begin{array}{c} n=\frac{N}{1+Ν*e^{2}}=400, \end{array}$$

where *n* = the sample size of the research; *N* = total number of smallholders; *e* = maximum variability or margin of error 5% (0.05); and 1 = the probability of the event occurring. Therefore, a total of 400 households were selected at 5% standard error with 95% confidence interval.

### 2.3. Data Collection Method

A multistage sampling technique was employed to select the study districts and their respective kebeles. From each district, three kebeles were purposively selected based on their sheep population and accessibility for transportation. Purposive sampling technique was used to select the households based on sheep production practices. Then, the list of households with minimum number of two sheep and had prior experience in sheep production was collected according to Kenfo et al. [5]. Sheep producers were randomly selected at household level for face-to-face interview, and 18 key informants (veterinarians, para veterinarians (agricultural development agents), and heads of districts' livestock development agency) were selected for in depth interview using a purposive sampling technique based on their rich experience. Both qualitative and quantitative data were used for this study, and structured and semi-structured questionnaire was initially designed and developed in English and translated into local language, Afan Oromo, and then back to English to check for consistency and clarity. The questionnaire was pretested before the interview on five purposively selected sheep producers for its clarity, palatability, and time efficiency so that it would not be boring for the interviewee. Then, the questionnaire was revised and used as a tool of data collection for the final interview. It was used to collect information on socioeconomic characteristics, sheep diseases, and management practices. The data collections were carried out by the data collector, whereas the supervision was carried out by the researchers.

### 2.4. Physical Clinical Examination

A thorough clinical examination was undertaken on sheep suspicious of any clinical conditions, discomfort, and illness. All the necessary clinical parameters were taken; the sheep were carefully inspected and palpated for any typical clinical signs of sheep diseases like ovine pasteurellosis, actinomycosis, ovine brucellosis, salmonellosis, sheep pox, PPR, Orf, mange mites, tick infestation, liver flukes, and lung worm. During physical clinical examination, different signs were observed such as abortion, lameness, oral and interdigital vesicle formations, coughing, watery diarrhea, excessive salivation, bottle jaw, weight loss, and loss of appetite.

### 2.5. Data Management and Analysis

The data entry, organization, and summarization were performed on an excel spreadsheet (Microsoft office® excel 2007). For the analysis, SPSS version 23 (Armonk, NY : IBM Corp.) software was used. Descriptive statistics was employed to describe the frequency, mean values, and proportion (percentages) of respondents; Pearson's chi-square test was used to compare the proportions of observations among the different categories. A *p* value of <0.05 and 95% confidence interval were used to determine the statistical significance of an estimate.

## 3. Results

### 3.1. Sociodemographic Information

From a total of 400 sheep producers involved in the study, a majority (75.8%) of the interviewed households were male headed. The educational status of the respondents was 32.8%, 15.5%, 10%, 5.5%, 12%, and 24.3% for illiterate, read and write, grade 1–4, grade 5–8, grade 9–12, and diploma and above, respectively. For the majority (51.5%) of the respondents, their age falls between 30–44 years. A vast majority (62.3%) of the respondents had more than five years of sheep production experience (Table 1).

### 3.2. Production and Management Practices

The mean number of sheep produce per household was 2.62 in the study areas. Extensive sheep production system was most practiced; (66.3%), (5.75%), and (2.50%) in the highland, midland, and lowland agroecologies, respectively. The respondents indicated that sheep have major role in highland (58.8%), midlands (6.0%), and lowlands (2.50%) for the livelihood of community in study areas. Separate pen housing system was used by the majority (59.8%) of sheep producers in highland agroecology. The design of sheep house was medium (34.5%) which was bedded using sand floor (79.8%) and grass (5.75%). Most of the drainage system of the house was poorly designed (46.8%), (3.0%), and (1.50%) in highland, midland, and lowland agroecology, respectively. The respondents were also indicated that free grazing in open environment was practiced in highlands (34.5%). The source of water was from pipe water (25.3%) for the animal in study areas (Table 2).

### 3.3. Major Sheep Diseases and Disease Conditions

The respondents indicated bacterial, viral, and parasitic diseases were the major sheep diseases in the study areas. According to the respondents, pasteurellosis was the major bacterial disease in highlands (55.8%), midlands (4.25%), and lowlands (1.0%). They have also shown that sheep pox was the major viral disease in highlands (54.5%), midlands (4.75%), and lowlands (1.0%). Mange mites were the major parasitic disease in highlands (41.3%), midlands (2.75%), and lowlands (1.0%). With regards to the disease conditions, loss of appetite was the most dominant abnormality occurred in highlands (66.8%), midlands (5.75%), and lowlands (1.25%), followed by weight loss in highlands (43.3%), midlands (10.6%), and lowlands (1.25%) (Table 3).

Besides, majority (71.3%) of the study participants have replied that, in all the three agroecologies, disease is the dominant factor which deteriorates the productivity of sheep in North Shewa Zone and summer (the cold rainy season in Ethiopia) is the most problematic season which exacerbates the occurrence of sheep diseases in the area (Table 4).

### 3.4. Management of Sheep Diseases

The finding of the present study has revealed that, majority of the farmers in highlands (67.3%), midlands (6.75%), and lowlands (2.50%) were treating their sheep using traditional medicines. The farmers treated the diseased sheep by taking into a private veterinary clinic in highlands (77.3%), midlands (6.0%), and lowlands (2.50%). The respondents were also indicated that the vaccination practice was very low in highlands and midlands with similar percentage (5.0%), but not in lowlands (Table 5).

## 4. Discussion

The present study has identified major sheep diseases, management practices, and veterinary service delivery in North Shewa Zone, Oromia Regional State of Ethiopia. This result was in line with other findings which were conducted in Western Tigray and Metekel Zone of Benishangul Gum Regional State, Northwestern Ethiopia; [2, 9, 14], which were identified major health challenges of sheep and husbandry problems in the areas in a similar fashion.

Bacterial, viral, and parasitic diseases were the major sheep diseases in which ovine pasteurellosis was a serious bacterial disease in highlands. The result also indicated that sheep pox was the major viral disease in highlands. Mange mites were the major parasitic disease followed by tick infestation. The finding was in line with the report of Fentie et al. [6] that was conducted in Amhara Region, Ethiopia. It was also analogous to Urgessa et al. [15] which was conducted in Ilu Abba Bora zone of Oromia Regional State, Southwestern Ethiopia.

The results of the study have also shown poor sheep management practices in the area. This was in line with the findings of Ferede et al. [16], a study conducted in selected model sheep villages of South Gondar Administrative Zone, Ethiopia. The study also indicated that the design of the sheep house was medium in the study areas, and it disagrees with the finding of Welay et al. [2] who reported a poor housing design. In the study area, the extensive sheep production system in which free grazing in an open environment in search of adequate feed where different species of grasses and crop residues and water are accessible is a common practice. This finding was also in agreement with the finding of Alilo et al. [17] and Jones et al. [18].

In this study, shortages of feed have been reported by the majority of respondents. This was also in line with the findings of Kenfo et al. [5], in Bensa district of Sidama Zone in Southern Ethiopia. This is primarily due to shortage of grazing land and farming expansion.

This study also revealed a noticeable effect of summer season on the occurrence of sheep disease in the study area. This might be due to the stressing effect of Ethiopian summer season which is too cold and rainy. Due to the cold stress, the immunity of sheep will be compromised and the animals are easily susceptible to microbial pathogens of major concern. The result is in agreement with the finding of Jones et al. [18], who reported the association of disease occurrence with malnutrition and adverse weather.

As far as the practice of treating a sick sheep is concerned, farmers usually lately take the sick sheep to veterinary clinics due to different challenges, including traditional beliefs that is awaiting the sick animal for self-recovery, inability to afford treatment costs, preference of taking to traditional healers, and treating by themselves using traditional medicine due to long distance to veterinary clinics. This finding also agrees with the finding of Maass et al. [19], Ferede et al. [16], and Pushpangadan et al. [20], all of which were suggested less accessibility of farmers to veterinary services due to economic problems, traditional taboos, and long distance between farmers' residence and veterinary clinics.

The results of the in-depth key informant interview have triangulated with the qualitative data why most farmers were not taking their animals to veterinary service center, prefer taking to traditional healers and treat by themselves. It revealed a socioeconomic and traditional practice demanding technical interventions. This finding was also in agreement with the finding of Welay et al. [2].

### 4.1. Limitation of the Study

The scope of the study was limited to six districts, this may fail to represent the whole North Shewa Zone. A cross-sectional study design was employed, but it is impossible to see a causal relationship by using this design [21]. The study relies on participants' response rates and physical clinical examination for reporting the major diseases and management practice,; no laboratory confirmation of the disease was undertaken. In addition, self-report by respondents was used during disease surveillances, which might be biased.

## 5. Conclusions

The study concluded that a high burden of sheep diseases was found in the area. There were poor management practices provided in the study areas which hindered sheep production and productivity. This indicated that designing effective prevention and control strategies and improving the management practices are crucial. Moreover, awareness creation should be done on sheep producers concerning major sheep diseases and improved management practices.

## Figures and Tables

**Table 1:** Sociodemography of the study participants in North Shewa Zone, Oromia, Ethiopia.

Variables		No.	Percent (%)
Sex	Male	303	75.8
	Female	97	24.3

Age	19–29	48	12.0
	30–44	206	51.5
	45–59	123	30.8
	>60	23	5.8

Educational status	Illiterate	131	32.8
	Read and write	62	15.5
	Grade 1–4	40	10.0
	Grade 5–8	22	5.5
	Grade 9–12	48	12.0
	Diploma and above	97	24.3

Sheep-producing experience	<2 yrs	47	11.8
	2–5 yrs	104	26.0
	>5 yrs	249	62.3

Agroecology	Highland	358	89.5
	Midland	32	8.0
	Lowland	10	2.5

Districts	Kimbibit	60	15.0
	Wuchale	67	16.8
	Degem	68	17.0
	Kuyu	67	16.8
	Jidda	70	17.5
	Girar Jarso	68	17.0

**Table 2:** Management practices of sheep in North Shewa Zone, Oromia, Ethiopia.

Variables		Agroecology		
		Highland	Midland	Lowland
		No. (%)	No. (%)	No. (%)
Production system	Intensive	50 (12.5)	2 (0.50)	—
	Semi-intensive	43 (10.8)	7 (1.80)	—
	Extensive	265 (66.3)	23 (5.75)	10 (2.5)

Role of sheep in livelihood	Major	235 (58.8)	24 (6.0)	10 (2.5)
	Minor	108 (27.0)	5 (1.25)	—
	No role	15 (3.75)	3 (0.75)	—

Housing system	Separate pen	239 (59.8)	18 (4.50)	—
	With other animals	89 (22.3)	14 (3.50)	5 (1.25)
	With human	30 (7.5)	—	5 (1.25)

Housing design	Good	119 (29.8)	10 (2.50)	5 (1.25)
	Medium	138 (34.5)	10 (2.50)	5 (1.25)
	Poor	101 (25.3)	12 (3.0)	—

Materials used for bedding	Grass	23 (5.75)	3 (0.75)	1 (0.25)
	Straw	16 (4.0)	6 (1.50)	—
	Sand	319 (79.8)	23 (5.75)	9 (2.25)

Stock density	Enough to turn around	112 (28.0)	10 (2.5)	2 (0.5)
	Enough to stand and lie down	207 (51.8)	13 (3.25)	8 (2.0)
	Unsuitable to turn around	39 (9.75)	9 (2.25)	—

Status of the drainage	Good	77 (19.3)	13 (3.25)	2 (0.5)
	Medium	94 (23.5)	7 (1.75)	2 (0.5)
	Poor	187 (46.8)	12 (3.0)	6 (1.5)

Types of feed	Free grazing	289 (72.3)	22 (5.5)	8 (2.0)
	Hay	32 (8.0)	10 (2.5)	2 (0.5)
	Concentrate	37 (9.25)	—	—

Frequency of watering	Once	201 (50.3)	8 (2.0)	—
	Twice	134 (33.5)	14 (3.5)	8 (2.0)
	Three times	18 (4.5)	10 (2.5)	2 (0.5)
	Four times	5 (1.25)	—	—

Source of water	Pipe water	94 (23.5)	5 (1.25)	2 (0.5)
	Pond water	42 (10.5)	—	5 (1.25)
	River	222 (55.5)	27 (6.75)	3 (0.75)

**Table 3:** Major sheep diseases and disease conditions in North Shewa Zone, Oromia, Ethiopia.

Variables		Agroecology		
		Highland	Midland	Lowland
		No. (%)	No. (%)	No. (%)
Bacterial diseases	Ovine pasteurellosis	223 (55.8)	17 (4.25)	4 (1.0)
	Actinomycosis	80 (20.0)	8 (2.0)	5 (1.25)
	Ovine brucellosis	23 (5.74)	3 (0.74)	–
	Salmonellosis	10 (2.50)	3 (0.75)	–
	Anthrax	22 (5.50)	1 (0.25)	1 (0.25)

Viral diseases	Sheep pox	218 (54.5)	19 (4.75)	4 (1.0)
	Pestis des ruminates	86 (21.5)	8 (2.0)	5 (1.25)
	Orf	21 (5.25)	3 (0.75)	–
	Blue tongue	33 (8.25)	2 (0.50)	1 (0.25)

Parasitic diseases	Mange mites	165 (41.3)	11 (2.75)	4 (1.0)
	Ticks	91 (22.8)	7 (1.75)	5 (1.25)
	Liver flukes	33 (8.25)	5 (1.25)	–
	Lung worm	69 (17.3)	9 (2.25)	1 (0.25)

Body condition score	Poor	92 (23.0)	3 (0.75)	–
	Medium	221(55.3)	23 (5.75)	10 (2.50)
	Good	33 (8.25)	3 (0.75)	–
	Fat	12 (3.0)	3 (0.75)	–

Major disease conditions	Wound	43 (10.8)	–	–
	Lameness	130 (32.5)	11 (2.75)	5 (1.25)
	Hoof overgrowing	12 (3.0)	–	–
	Weight loss	173 (43.3)	21 (10.6)	5 (1.25)

Feeding and drinking behavior	Loss of appetite	267 (66.8)	23 (5.75)	5 (1.25)
	Normal	91 (22.8)	9 (2.25)	5 (1.25)

**Table 4:** Respondents' view on the seasonal occurrence of sheep disease in North Shewa Zone.

Variables		Response rate	
		Number of respondents	Percent
Season	Summer	285	71.3
	Winter	63	15.8
	Autumn	19	4.8
	Spring	33	8.1

Total		400	100

**Table 5:** Veterinary services of sheep in North Shewa Zone, Oromia, Ethiopia.

Variables		Agroecology		
		Highland	Midland	Lowland
		No. (%)	No. (%)	No. (%)
Do you treat the diseased sheep?	Yes	270 (67.5)	16 (4.0)	8 (2.0)
	No	88 (22.0)	16 (4.0)	2 (0.5)

Stages of treatment	Early	84 (21.0)	14 (3.5)	5 (1.25)
	Lately	269 (67.3)	16 (4.0)	5 (1.25)
	Never	5 (1.25)	2 (0.5)	—

Who treats the diseased sheep?	Take to a veterinary clinic	264 (66.0)	32 (8.0)	10 (2.50)
	Treat myself	68 (17.0)	—	—

Types of veterinary services	Government	49 (12.3)	8 (2.0)	10 (2.50)
	Private	309 (77.3)	24 (6.0)	26 (6.50)

Treatment practices	Supportive treatment	6 (1.50)	—	—
	Modern drugs	83 (20.8)	5 (1.25)	—
	Traditional medicine	269 (67.3)	27 (6.75)	10 (2.50)

Purpose of treatment	As deworming	185 (46.2)	—	—
	As vaccination	20 (5.0)	20 (5.0)	—
	Apparent are observed	19 (4.75)	—	2 (0.5)
	Severely sicken	55 (13.8)	3 (0.75)	5 (1.25)
	Regular checkup of the herd	79 (19.8)	9 (2.25)	3 (0.75)

## Data Availability

The dataset used in this study is available on reasonable request from the corresponding author.
